# Jinwujiangu Capsule Treats Fibroblast-Like Synoviocytes of Rheumatoid Arthritis by Inhibiting Pyroptosis via the NLRP3/CAPSES/GSDMD Pathway

**DOI:** 10.1155/2021/4836992

**Published:** 2021-11-22

**Authors:** Yi Ling, Mao Xiao, Zhao-Wei Huang, Hui Xu, Fang-Qin Huang, Ni-Na Ren, Chang-Ming Chen, Dao Min Lu, Xue-Ming Yao, Li-Na Xiao, Wu-Kai Ma

**Affiliations:** ^1^The Second Affiliated Hospital of Guizhou University of Traditional Chinese Medicine, Guiyang 550000, Guizhou Province, China; ^2^Guizhou Anshun People's Hospital, Anshun 561000, Guizhou Province, China

## Abstract

Jinwujiangu capsule (JWJGC) is a traditional Chinese medicine formula used to treat rheumatoid arthritis (RA). However, whether its mechanism is associated with pyroptosis remains unclear. In this study, the ability of JWJGC to inhibit the growth of fibroblast-like synoviocytes of RA (RA-FLS) through pyroptosis was evaluated. The cells isolated from patients with RA were identified by hematoxylin and eosin (H&E) staining, immunohistochemistry, and flow cytometry. After RA-FLS were treated with different concentrations of JWJGC-containing serum, the cell proliferation inhibition rate, expression of caspase-1/3/4/5, NOD-like receptor protein 3 (NLRP3), gasdermin-D (GSDMD), and apoptosis-associated speck-like protein containing a CARD (ASC), concentrations of interleukin-1*β* (IL-1*β*) and interleukin-18 (IL-18), the activity of lactic dehydrogenase (LDH), and pyroptosis were evaluated. The results showed that JWJGC increased the proliferative inhibition rate, decreased the expression of caspase-1/3/4/5, GSDMD, NLRP3, and ASC, suppressed the expression of IL-1*β* and IL-18, induced the activity of LDH, and downregulated the number of double-positive FITC anti-caspase-1 and PI. Generally, our findings suggest that JWJGC can regulate NLRP3/CAPSES/GSDMD in treating RA-FLS through pyroptosis.

## 1. Introduction

Rheumatoid arthritis (RA) is a chronic disease characterized by autoimmune functional disorders caused by genetic and environmental factors, which results in hyperplasia of synovial membranes, formation of vascular pannus, and destruction of the cartilage and bone [1]. Currently, the underlying mechanism of RA is not completely understood. However, it has been found that the synovial tissue of RA shows tumor-like growth characteristics accompanied by a high concentration of inflammatory factors related to the development of RA [2]. Fibroblast-like synoviocytes (FLS) constitute the main part of the synovial tissue and play an important role in the pathogenesis of RA [3]. Therefore, it is beneficial for RA patients to inhibit the migration of FLS and the expression of inflammatory factors.

Pyroptosis is programmed cell death initiated by inflammasomes [4]. Although pyroptosis can protect the host from microbial pathogens, its dysregulation leads to a variety of autoimmune and autoinflammatory conditions. After stimulating the compromised state signal originating from the host due to bacteria, fungi, and parasites, inflammasomes have different pattern-recognition receptors (PRRs) such as melanoma 2 (AIM2), pyrin, and NOD-like receptor protein 3 (NLRP3) [5]. Inflammasomes drive the activation of caspases, including caspase-1/3/4/5. On one hand, the activated caspase-1 promotes prointerleukin-1*β* (pro-IL-1*β*) and prointerleukin-18 (pro-IL-18) to be mature interleukin-1*β* (IL-1*β*) and interleukin-18 (IL-18), which can both trigger an inflammatory reaction [6]. On the other hand, activated caspase-1 cleaves gasdermin-D (GSDMD) to separate its N-terminal pore-forming domain (PFD) from the C-terminal repressor domain (RD). PFD works on the membrane of cells to form pores that drive swelling and membrane rupture [7]. Meanwhile, inflammatory storms occur after IL-1*β* and IL-18 are released into the extracellular space.

**Figure 1:**
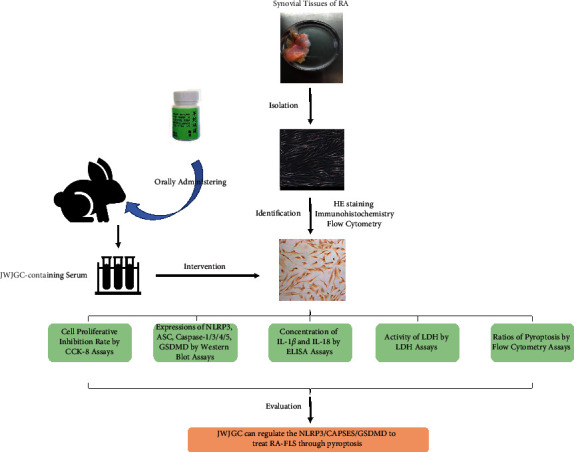
The work flowchart of this study.

Jinwujiangu capsule (JWJGC) is a hospital formula developed by Professor Wu-Kai Ma based on the theory of Miao medicine. In the past 20 years, this Chinese medicine prescription has been effective in improving the clinical symptoms of patients with few RA side effects. In previous studies, JWJGC decreased the ratio of joint swelling in a rat model and inhibited cell proliferation with low levels of inflammatory cytokines, including IL-1*β* and IL-18 in RA-FLS [8]. However, whether its mechanism of action is associated with pyroptosis remains unclear. The present study aimed to evaluate the regulatory effect of JWJGC on RA-FLS through pyroptosis via the NLRP3/CAPSES/GSDMD pathway. The flowchart of the study procedure is shown in Figure 1.

## 2. Materials and Methods

### 2.1. RA-FLS Isolation

We followed the methods described by Yi Ling et al. [9]. RA-FLS cells were isolated and cultured using the explant adherent culture method. Synovial tissue samples were obtained from three patients (two men and one woman: 0–70 years) during joint replacement surgery at the Second Affiliated Hospital of Guizhou University of Traditional Chinese Medicine. All patients were diagnosed with RA according to the 2010ACR/EULAR Classification Criteria for Rheumatoid Arthritis [10]. The experiments were approved by the Medical Ethical Committee of the Second Affiliated Hospital of Guizhou University of Traditional Chinese Medicine (PY2019104), and all patients provided written informed consent. The synovial tissues were washed five times with phosphate-buffered saline (PBS; 0626A18, BI, Israel) supplemented with 2% penicillin-streptomycin (J180027, HyClone, USA). After removing irrelevant tissues, the synovial tissue was cut into approximately one cubic centimeter pieces. All pieces were seeded in a 25 cm^2^ culture flask for anchorage without a culture medium at 37°C in a humidified atmosphere of 5% CO_2_ for 4 h. Then, 5 mL of the complete medium containing Dulbecco's modified Eagle's medium (DMEM; 8119424, Gibco, USA) supplemented with 20% fetal bovine serum (1948791, BI, Israel), and 1% penicillin-streptomycin (J180027, HyClone, USA) was added to the culture flask to harvest RA-FLS after approximately 3 weeks. Cells were subcultured at a ratio of 1 : 2 when they reached 90% confluence, and the cells at passages 3–6 were used for the experiments.

### 2.2. Cell Identification

We followed the methods described by Yi Ling et al. for cell identification [9]. Hematoxylin and eosin (H&E) staining and immunohistochemistry were used to identify RA-FLS. The third passage cells (3.5 × 10^5^ cells/mL) were seeded on 10 mm × 10 mm coverslips in a 6-well plate for 24 h. After the cells were fixed on coverslips with 4% paraformaldehyde for 30 min, they were stained as per the manufacturer's instructions using an H&E staining kit (20190507, Solarbio, China). The fixed cells on coverslips were incubated with the primary rabbit antivimentin antibody (1 : 250; ab92547, Abcam, UK) at 37°C overnight. Horseradish peroxidase (HRP)-conjugated goat anti-rabbit IgG antibody (K195219C, ZSGB, China) served as the secondary antibody, and then, chromogen development was obtained with 3,3-diaminobenzidine reagent (K193328E, ZSGB, China). Subsequently, the FLS were counterstained with hematoxylin.

Flow cytometry was used to determine the purity of RA-FLS staining for the cell surface markers CD90 or VCAM-1. The third passage FLS (1 × 10^5^ cells/mL) suspended in 1 mL of PBS in 12 × 75 mm^2^ tubes were centrifuged at 200 × g for 5 min at 4°C. The supernatant was then removed. The cells were incubated 30 min after 5 *μ*L of FITC anti-CD90 (ab11155, Abcam, UK) or APC anti-VCAM-1 (ab103173, Abcam, UK) was added in the dark at 4°C. After three times of washing, the cells were resuspended in 500 *μ*L of a buffer solution and analyzed using a flow cytometer (FACSCanto II, BD, USA).

### 2.3. Preparation of Drug-Containing Serum

#### 2.3.1. Experimental Drug of JWJGC

JWJGC consisting of *Gardneria angustifolia* Wall. 10 g, *Zaocys dhumnades* 10 g, Curcumae Longae Rhizoma 15 g, Caulis Sinomenii 15 g, Paeoniae Radix Alba 15 g, Cibotii Rhizoma 15 g, *Homalomena occulta* 10 g, *Panax notoginseng* (Burk.) F. H. Chen 5 g, and licorice 3 g. JWJGC (batch number: 20181001) was obtained from the Second Affiliated Hospital of Guizhou University of Traditional Chinese Medicine. Drug quality control was also available.

#### 2.3.2. Experimental Animals and Feeding Conditions

Nine male and nine female, healthy New Zealand White rabbits (3 ± 0.5 kg) were supplied by the Animal Experimental Center of Guizhou Medical University (batch number: SCXK (Guizhou) 2018-0001) and were housed in a standard 12-h light/dark cycle with water and food ad libitum at 20–25°C for 7 days. This experimental program was approved by the experimental animal ethics committee (ethical approval code: GK2019004).

#### 2.3.3. Experimental Serum

Nine times the equivalent dose for rabbits was 3.645 g/1000 g of JWJGC according to the calculation by weight ratio between humans and rabbits, while 3.75 mg/1000 g of leflunomide (batch number: 190101, Suzhou Changzheng-Xinkai Pharmaceutical Co., Ltd., China) was the positive control. The rabbits were randomly divided into three groups, each having three males and three females. JWJGC (3.645 g/1000 g), leflunomide (3.75 mg/1000 g), and an equal volume of distilled water were orally administered to the rabbits twice daily at 9:00 am and 9:00 pm for 5 days. The rabbits were sacrificed by air injection through the auricular vein 2 h after the final administration. Blood was collected from the hearts of the rabbits under aseptic conditions. The serum was separated by centrifugation at 3000 rpm for 20 min, inactivated at 56°C for 30 min, sterilized with 0.22 *μ*m millipore filters, and stored separately at −20°C for the follow-up study. The dose per mg administered to the rabbits was calculated as follows: M_1_ = M_2_ × 3.08, where M_1_ is the dose per kg rabbit per day and M_2_ is the dose per kilogram of adults per day.

#### 2.3.4. RA-FLS Treatments

The cells were divided into six groups according to the treatment. The blank serum control group was treated with DMEM supplemented with 20% fetal bovine serum and 1% penicillin-streptomycin, whereas the rabbit serum control group was treated with DMEM supplemented with 20% rabbit serum and 1% penicillin-streptomycin. The JWJGC low-dose group (JWJGC-L) was treated with DMEM supplemented with 5% JWJGC-containing serum, 15% rabbit serum, and 1% penicillin-streptomycin. The JWJGC medium-dose group (JWJGC-M) was treated with DMEM supplemented with 15% JWJGC-containing serum, 5% rabbit serum, and 1% penicillin-streptomycin. The JWJGC high-dose group (JWJGC-H) was treated with DMEM supplemented with 20% JWJGC-containing serum and 1% penicillin-streptomycin. The leflunomide-positive control group was treated with DMEM supplemented with 20% leflunomide-containing serum and 1% penicillin-streptomycin.

### 2.4. Cell Counting Kit-8 (CCK-8) Assay

The CCK-8 assay (CCK-8; NW595, Dojindo, Japan) was used to detect the cell proliferative inhibition rate according to the manufacturer's protocol. Briefly, the third passage cells were seeded in 96-well plates (6 × 10^3^ cells/well) and incubated at 37°C and 5% CO_2_ for 24 h. After the cells were subjected to 180 *μ*L of different treatments for 24 h or 48 h, 20 *μ*L CCK-8 solution was added to each well and further incubated for 2 h at 37°C. The absorbance values were measured at a wavelength of 450 nm using a microplate reader (1510, Thermo Fisher Scientific, Waltham, MA, USA). Cell proliferative inhibition rate = (OD value of contrast well − OD value of experimental well)/(OD value of contrast well − OD value of blank well) × 100%.

### 2.5. Western Blot Assays

The third passage cells (1.2 × 10^6^ cells) underwent different treatments in a 75 cm^2^ culture flask for 24 h. The supernatants were preserved at −20°C for the succeeding assays. Total proteins were extracted from cells with RIPA lysis buffer (20170510, Solarbio, China), followed by quantification using a BCA protein concentration determination kit (20190921, Solarbio, China) according to the manufacturer's protocol. Cell lysates containing 40 *μ*g total protein were loaded onto 10% sodium dodecyl sulfate-polyacrylamide gel electrophoresis gels (1610185, Bio-Rad, USA). The separated protein bands were transferred onto nitrocellulose membranes. After blocking with 5% skim milk in Tris-buffered saline-Tween (TBST) for 1.5 h, membranes were incubated overnight at 4°C with different primary antibodies, including those against NLRP3 (ab210491, Abcam, UK; 1 : 1000), caspase-1 (ab62698, Abcam, UK; 1 : 500), caspase-3 (ab13847, Abcam, UK; 1 : 500), caspase-4 (ab238124, Abcam, UK; 1 : 1000), caspase-5 (ab40887, Abcam, UK; 1 : 1000), GSDMD (ab210070, Abcam, UK; 1 : 1000), and apoptosis-associated speck-like protein containing CARD (ASC) (ab151700, Abcam, UK; 1 : 1000). The membranes were washed five times with TBST, and then, peroxidase-conjugated goat anti-rabbit IgG antibody (019189, Pumei, China; 1 : 10000) was used as a secondary antibody and incubated for 1 h. After five more washes with TBST, an enhanced chemiluminescence substrate (170-5060, Bio-Rad, USA) was used to detect the amount of target proteins. ImageJ software was used to quantify the relative intensity following normalization with *β*-actin (4970S, Cell Signaling, USA; 1 : 1,000).

### 2.6. ELISA Assays

The supernatants originated from “2.5” thawed at room temperature, and concentrations of IL-1*β* (ab214025, Abcam, UK) and IL-18 (ab215539, Abcam, UK) were determined by ELISA kits according to the manufacturer's protocol.

### 2.7. LDH Assays

Lactic dehydrogenase (LDH), a key feature of cells undergoing apoptosis, necrosis, and other forms of cellular damage, is rapidly released into the cell culture supernatant when the plasma membrane is damaged. The activity of LDH was tested using LDH assay kits (20200630, Nanjing Jiancheng Bioengineering Institute, China) according to the manufacturer's instructions. From “2.5,” 25 *μ*L of supernatant was moved to a 96-well plate, and 25 *μ*L of reconstituted substrate mix was added to each well. The plate was then incubated for 15 min in a dark room at 37°C. Then, 25 *μ*L of 2,4-dinitrophenylhydrazine was added to each well and incubated for 5 min in a dark room at 37°C. Moreover, 250 *μ*L NaOH (0.4 mol/L) was added to each well and incubated for another 5 min in a dark room at 37°C. Absorbance was recorded at 450 nm using a microplate reader (1510, Thermo Fisher Scientific, Waltham, MA, USA). Activity of LDH (U/L) = (OD value of experimental well − OD value of contrast well)/(OD value of standard well − OD value of blank well) × 0.2 *μ*M.

### 2.8. Flow Cytometry Assays

After the third passage cells (1.2 × 10^6^ cells) underwent different treatments, the cells were incubated in 5 *μ*L of FITC anticaspase-1 (SC-392736, Santa Cruz Biotechnology, USA) in a dark room at 37°C for 1 h, washed with 2 mL of apoptosis buffer, and centrifuged. Then, 5 *μ*L of PI was added to the tube, followed by another incubation for 15 min. Consequently, the cells were resuspended in 500 *μ*L of apoptosis buffer and analyzed by flow cytometry (FACSCanto II, BD, USA).

### 2.9. Statistical Analysis

Statistical analysis was performed using the SPSS 17 software program (SPSS Inc., USA). The one-way analysis of variance was used to compare the means of the different groups. Differences were considered statistically significant at *P* < 0.05.

## 3. Results

### 3.1. H&E Staining, Immunohistochemistry, and Isolation Purity

**Figure 2:**
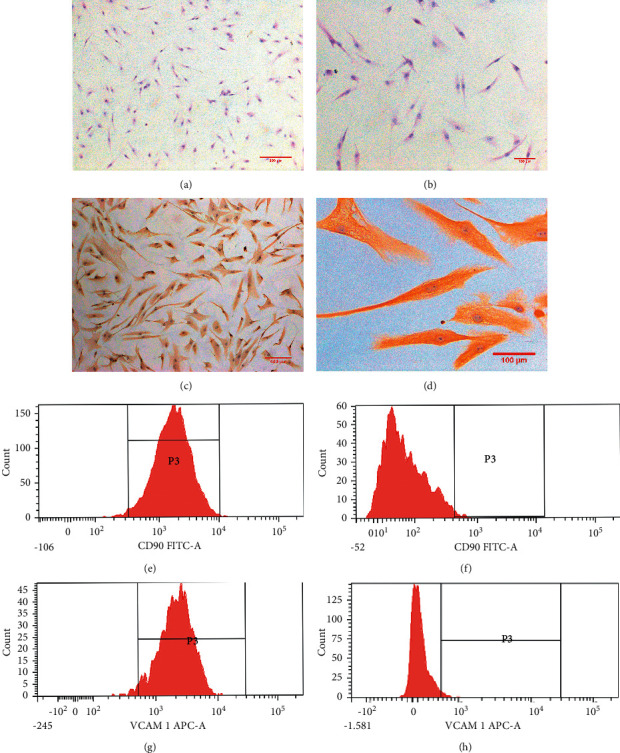
(a) (×100) and (b) (×200) showing the H&E staining results. Immunohistochemistry results are shown in (c) (×100) and (d) (×200). (e) The purity of the isolated cells marked with FIDC anti-CD90 (96.6 ± 1.44%; *n* = 4). (f) The control. (g) The purity of the isolated cells marked with APC anti-VCAM-1 (97.1 ± 0.39%; *n* = 4). (h) The control.

The third passage RA-FLS suffered from H&E staining and immunohistochemistry after being purified by passage culture. The cytoplasm was red and the nucleus was blue in H&E staining (Figures 2(a) and 2(b)), while the cells were spindle-shaped with blue nuclei and brown cytoplasm in immunohistochemistry (Figures 2(c) and 2(d)). Immunohistochemistry results indicated that vimentin was positive. Tissue origin, cell shape, and positive immunohistochemistry results proved that the observed cells were RA-FLS. The purity of the isolated cells was 96.6 ± 1.44% (*n* = 4) marked with FITC anti-CD90 or 97.1 ± 0.39% (*n* = 4) with APC anti-VCAM-1 (Figures 2(e), 2(f), 2(g), and 2(h)).

### 3.2. JWJGC Affected Cell Proliferation

**Figure 3:**
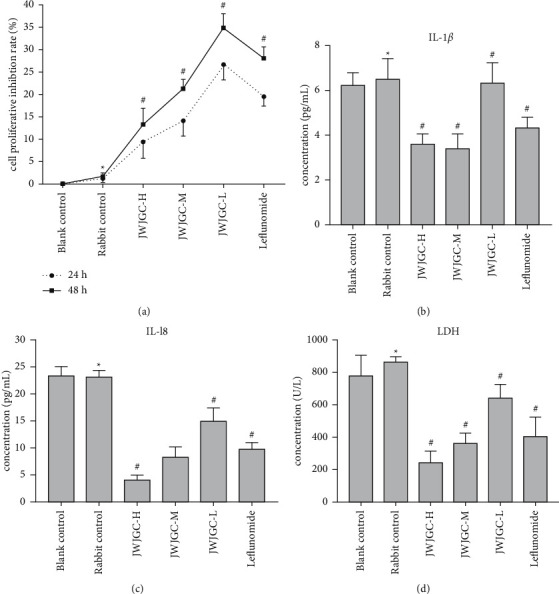
(a) The cell proliferative inhibition rate after the different treatments for 24 h or 48 h. (b)-(c) The concentrations of IL-1*β* and IL-18. (d) The concentrations of LDH. Data are expressed as mean ± SEM (*n* = 5). ^*∗*^*P* > 0.05, compared with blank control; ^#^*P* < 0.05, compared with the rabbit group. Blank control, blank serum control group; rabbit control, rabbit serum control group; JWJGC-H, JWJGC high-dose group; JWJGC-M, JWJGC medium-dose group; JWJGC-L, JWJGC low-dose group; Leflunomide, leflunomide-positive control group.

Compared with the blank serum control group, there was no significant difference in the cell proliferative inhibition rate in the rabbit serum control group (*P* > 0.05). However, it was significantly elevated in the JWJGC-L, JWJGC-M, JWJGC-H, and leflunomide-positive control groups, relative to the rabbit serum control group (*P* < 0.05). Moreover, the cell proliferative inhibition rate was proportional to the concentration of JWJGC-drug serum and the time of the intervention (Figure 3(a)).

### 3.3. JWJGC Affected the Expressions of NLRP3, ASC, Caspase-1/3/4/5, and GSDMD

**Figure 4:**
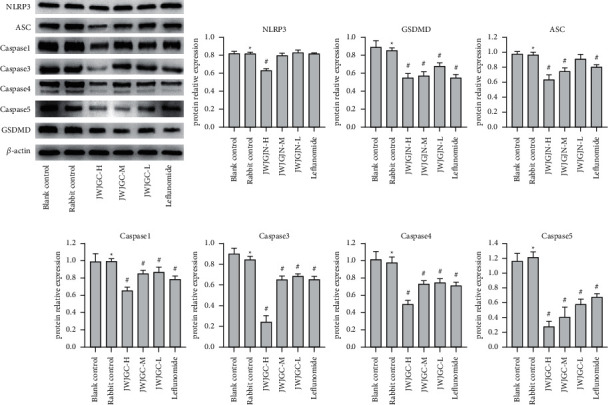
Western blot bands of the expression of NLRP3, ASC, GSDMD, and caspase-1/3/4/5 after the different treatments. Data are expressed as mean ± SEM (*n* = 3). ^*∗*^*P* > 0.05 compared with blank control; ^#^*P* < 0.05 compared with the rabbit group. Blank control, blank serum control group; rabbit control, rabbit serum control group; JWJGC-H, JWJGC high-dose group; JWJGC-M, JWJGC medium-dose group; JWJGC-L, JWJGC low-dose group; Leflunomide, leflunomide-positive control group.

There was no significant difference in the expression of NLRP3, ASC, caspase-1/3/4/5, and GSDMD between the blank serum control group and the rabbit serum control group (*P* > 0.05). However, compared with the rabbit serum control group, caspase-1/3/4/5 and GSDMD displayed decreased expression in the JWJGC-L, JWJGC-M, JWJGC-H, and leflunomide-positive control groups (*P* > 0.05), NLRP3 expression was reduced only in the JWJGC-H group, and ASC declined in the JWJGC-M, JWJGC-H, and leflunomide-positive control groups (*P* < 0.05) (Figure 4).

### 3.4. JWJGC Affected IL-1*β* and IL-18 Secretion

JWJGC did not affect the secretion of IL-1*β* and IL-18 after treatment in both the blank and rabbit serum control groups (*P* > 0.05). Nevertheless, the concentrations of IL-1*β* and IL-18 were both lower in JWJGC-L, JWJGC-M, JWJGC-H, and leflunomide-positive control groups than those of the rabbit serum control group (*P* < 0.05) (Figures 3(b) and 3(c)).

### 3.5. JWJGC Affected LDH Activity

The activity of LDH in the rabbit serum control group did not change significantly compared with that in the blank serum control group (*P* > 0.05). The activity of LDH in the JWJGC-L, JWJGC-M, JWJGC-H, and leflunomide-positive control groups was inhibited, compared with the rabbit serum control group (*P* < 0.05) (Figure 3(d)).

### 3.6. JWJGC Affected Pyroptosis in RA-FLS

**Figure 5:**
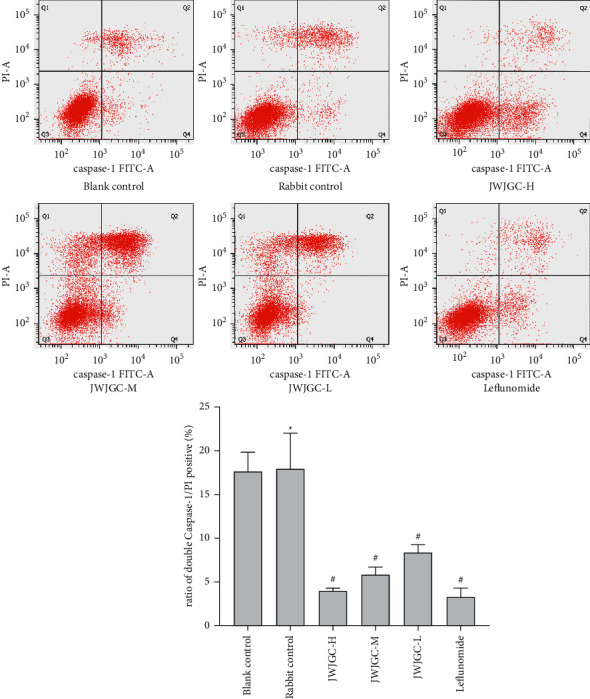
Double-positive caspase-1/PI is shown in the Q2 of every image. Data are expressed as mean ± SEM (*n* = 3). ^*∗*^*P* > 0.05 compared with blank control; ^#^*P* < 0.05 compared with the rabbit group. Blank control, blank serum control group; rabbit control, rabbit serum control group; JWJGC-H, JWJGC high-dose group; JJWJGC-M, JWJGC medium-dose group; JWJGC-L, JWJGC low-dose group; Leflunomide, leflunomide-positive control group.

The pyroptosis of the RA-FLS was marked with double positivity for FITC antcaspase-1 and PI, which was shown in the Q2 of every image. There was no significant difference between the blank and rabbit serum control groups (*P* > 0.05). However, the ratios of pyroptosis were significantly blocked in the JWJGC-L, JWJGC-M, JWJGC-H, and leflunomide-positive control groups, compared with the rabbit serum control group (*P* < 0.05) (Figure 5).

## 4. Discussion

RA is an autoimmune disease characterized by hyperplastic synovial tissue that eventually destroys the cartilage and bone. In its pathological process, the synovium is transformed from a relatively acellular structure to a hyperplastic and invasive tissue, and the lining layer expands from 1 to 2 cells to a depth of approximately 10–20 cells [11]. The resident cells in the synovial membrane lining layer are FLS (type B synovial cells) and synovial tissue macrophages (type A synovial cells) [12]. FLS are the target cells in the development of destructive joint inflammation of synovial tissue in RA with an overproduction of enzymes and infiltration of immune cells [13]. RA-FLS show unique invasive characteristics, which are autonomous and vertically transmitted, and these cells function as the main promoters of inflammation [14]. However, the ability of RA-FLS to proliferate in joints is limited, and its abnormal proliferation is brought about by the change in programmed cell death accompanied by increased expression of cytokines to some extent [15]. In addition, its ability to secrete cytokines, chemokines, and angiogenic factors is enhanced, and the unbalanced cytokine network leads to the enhancement of the inflammatory response, which eventually develops into RA [16]. In the present study, the cell proliferative inhibition rate changed with the time of intervention and concentrations of JWJGC-drug serum, indicating that JWJGC improved the tumor-like growth characteristics of RA-FLS to inhibit proliferation in the treatment of RA.

NLRP3 is a cytosolic signaling complex that mediates inflammatory responses and is often involved in the pathogenesis of noninfectious diseases. It responds primarily to danger signals, such as aging, overnutrition, or environmental changes [17]. It is mainly expressed in monocytes, macrophages, dendritic cells, and neutrophils; however, elevated levels of NLRP3 expression are also observed in RA patients [18]. ASC connects NLRP3 and procaspase-1, which is involved in the priming phase of pyroptosis [19]. Caspase-1 not only matures IL-1*β* and IL-18 but also induces membrane perforation through GSDMD [20]. Caspase-3/4/5 are unable to activate pro-IL-1*β* and pro-IL-18, but they can induce pyroptosis by GSDMD or GSDME [21,22]. In this study, the expression of NLRP3, caspase-1/3/4/5, ASC, and GSDMD declined after treatment with JWJGC-drug serum, implying that GWJGC could downregulate the expression of NLRP3, caspase-1/3/4/5, ASC, and GSDMD.

IL-1*β* is one of the primary explicit members of the IL-1 family and is produced by myeloid cells. It initiates innate immunity and forms an adaptive immune response during acute inflammation [23]. However, if IL-1*β* is overactivated, chronic oxidative stress, oxidative damage of DNA sequence, epigenetic changes, and autoimmune diseases may occur [24, 25]. IL-18 belongs to the IL-1 family and promotes the differentiation and production of cytokines based on specific receptors on the cell membrane [26]. For example, IL-18 mediates the expression of IL-1, TNF-*α*, and IL-6 via the NF-*κ*B pathway [27]. IL-1*β* and IL-18 are present in the cytoplasm at rest, without biological function; however, when caspases are activated by inflammasomes, IL-1*β* and IL-18 are activated. Most of the cytokines are secreted from cells through the endoplasmic reticulum to exert biological effects, while IL-1*β* and IL-18 cannot be released into the extracellular environment through the endoplasmic reticulum pathway because of the absence of an N-terminal secretion signal sequence. However, they can be transported outside with the assistance of the GSDMD protein related to perforation [28]. In these experiments, the concentrations of IL-1 and IL-18 were decreased by treatment with JWJGC-drug serum, indicating that JWJGC could reduce the secretion of IL-1 and IL-18.

Programmed cell death is the main mechanism of the cellular metabolism that maintains growth and immune homeostasis. Human programmed cell death patterns mainly include apoptosis, necrosis, autophagy, and pyroptosis, which are both morphological and biochemical [29]. Pyroptosis was discovered in the 1990s and was formally named in the early 21st century to distinguish it from cell necrosis and apoptosis [4]. Pyroptosis is divided into classical and nonclassical pathways according to the caspases [30]. In the classical pathway, inflammasomes are polymeric protein complexes composed of PRR, procaspase-1, and ASC, which activate caspase-1 [31–34]. Subsequently, active caspase-1 converts pro-IL-1*β* and pro-IL-18 into mature IL-1*β* and IL-18 [35]. Meanwhile, the N-terminus of GSDMD forms pores via active caspase-1. GSDMD p30 is localized to the lipid bilayer, whereas GSDMD p20 remains in the water environment. In liposomes, p30 exists as a high-level oligomer with a circular structure to recognize phospholipid molecules on cell membranes. Eventually, 18 nm channels were formed, which means pyroptosis occurred [36–38]. In the nonclassical pathway of pyroptosis, humans are mediated by caspase-3/4/5, and mice are mediated by caspase-11 [39, 40]. LDH activity reflects the content of the damaged cell membrane because LDH can only be released into the extracellular space through such membrane [41]. The results showed that the number of pyroptotic cells and the activity of LDH were lower with the treatment of JWJGC-drug serum, which demonstrated that GWJGC could prevent pyroptosis.

## 5. Conclusions

JWJGC inhibited the proliferation of RA-FLS by downregulating the expression of NLRP3, ASC, caspase-1/3/4/5, and GSDMD, reducing the secretion of IL-1*β* and IL-8, repressing the activity of LDH, and decreasing the double-positive FITC anticaspase-1 and PI. Collectively, our results demonstrate that JWJGC can treat RA-FLS by regulating pyroptosis via the NLRP3/CAPSES/GSDMD pathway. Further studies on the regulation of genes need to explore the therapeutic mechanisms underlying JWJGC effects in RA.

## Data Availability

The datasets used to support the findings of this study are available from the corresponding author upon request.
